# Development of a Cell-Based Assay to Assess Binding of the proNGF Prodomain to Sortilin

**DOI:** 10.1007/s10571-017-0558-1

**Published:** 2017-10-24

**Authors:** Ibrahim Malik, Søren Christensen, Jeffrey B. Stavenhagen, Gunnar P. H. Dietz

**Affiliations:** Department Neurodegeneration, H. Lundbeck A/S, Ottiliavej 9, 2500 Valby, Denmark

**Keywords:** Cellular assay, Cell-based assay, SORLA family, Nerve growth factor (NGF), Brain-derived neurotrophic factor (BDNF), Apoptosis

## Abstract

Sortilin was first identified based on its activity as part of intracellular protein sorting machinery. Recently, it was discovered that sortilin also acts as a cell surface receptor for the propeptide form of nerve growth factor (proNGF), progranulin, and neurotensin. The interaction of sortilin to these neurotrophic ligands is linked to diseases of the nervous system that lead to neurodegeneration and neuropathic pain. Blocking of the interaction of sortilin to these ligands may prevent or slow the progress of these nervous system disorders. In vitro screening assays for blocking compounds or peptides are part of the standard set of tools for drug discovery. However, assays for sortilin biology are not readily available to determine if the selected blocking agent inhibits sortilin activity on the surface of cells. We have developed a sortilin specific cell based assay to identify compounds that specifically block interaction between sortilin and proNGF prodomain. The assay system records both the presence of sortilin on the cell surface and the interaction with the pro domain of NGF. Fluorescent images of the sortilin expressing cells are analyzed for the presence of pro domain of NGF. Sortilin-positive and sortilin-negative cells within one well are concomitantly and automatically analyzed. Sortilin—pro domain interaction can be blocked dose dependently by neurotensin and synthetic compounds. The assay will facilitate the discovery of entities interfering with the binding of sortilin to the NGF pro domain. This assay can be modified to screen for inhibitors of the binding of ligands to other complex cell surface receptors.

## Introduction

Sortilin is a complex membrane bound receptor that plays an important role in the intra-cellular sorting of post translationally modified proteins. In addition to its intra-cellular role, sortilin is also expressed on the surface of neurons where it regulates neurotrophin signal transduction together with p75^NTR^ (Nykjaer et al. 2004). The tri-partite complex with proNGF and the p75^NTR^ is suggested to be involved in neuronal apoptosis in many nervous system diseases, including Alzheimer’s and Parkinson’s disease, seizure, spongiform encephalomyelopathy, retinal ischemia, spinal cord injury, ageing (Nykjaer and Willnow 2012), and neuropathic pain (Lewin and Nykjaer 2014). Signaling mediated by sortilin-ligand interaction has been implicated in the etiology of nervous system disorders. Sortilin is a receptor for neurotensin (Mazella et al. 1998), a 13-amino acid neuropeptide identified as a target for autism disorders (Patel et al. 2016) and obesity (Li et al. 2016). Sortilin was identified as receptor for progranulin (PGRN) (Hu et al. 2010; Zheng et al. 2011). Sortilin-mediated PGRN endocytosis leads to reduction in the level of circulating PGRN and may play a central role in the inheritable forms of frontotemporal lobar degeneration pathophysiology (Hu et al. 2010).

Sortilin is highly expressed in the human central nervous system, heart, placenta, skeletal muscle, testis, thyroids and other tissues (Petersen et al. 1997). Sortilin knockout mice are viable and only display a mild phenotype including a lessened sensitivity to pain (Devader et al. 2016; Zeng et al. 2009). Based on its broad expression profile, sortilin is potentially playing different roles in the tissues where it is expressed. The role of sortilin on neurons is not fully understood. This is underscored by studies that show sortilin-ligand interactions can promote neuronal apoptosis, but, by enhancing neurotrophin signaling, can also promote neuronal survival (Vaegter et al. 2011). Complex web of ligand interactions supports the need of a cell based assay that can dissect the roles of individual ligands.

The role of sortilin signaling in several pathologic processes makes it a target for drug development. Recently, high throughput screening efforts identified novel compounds AF40431 (Andersen et al. 2014) and AF48469 (Schrøder et al. 2014), which blocked sortilin-neurotensin interaction. These compounds were shown to inhibit neurotensin—sortilin binding using a scintillation proximity assay. In addition to this assay, other in vitro assays including the binding of ligands to membrane fractions derived from sortilin-expressing cells (Carvelli et al. 2017), surface plasmon resonance, and liposome flotation assays (Botta et al. 2009; Sparks et al. 2016) have been used to identify inhibitors of binding of ligands to sortilin. These data support the identification of hits from high throughput screen but fall short of predicting a functional role for these inhibitors blocking sortilin mediated cell signaling.

Reliable cellular assays are required to identify sortilin binding molecules that have therapeutic potential by blocking sortilin signaling pathways that lead to disease. A cellular assay using ^125^I-labeled proNGF (Clewes et al. 2008) and immunoprecipitation methods (Botta et al. 2009) to assess binding to cell surface expressed sortilin have been described, however, such assays are labor intensive and can only be used at a low throughput. In addition, a cell-based chemiluminescence proximity assays has been developed specifically for interactions with Amyloid precursor like protein 2 (Butkinaree et al. 2015). This assay is for a specific application that does not address the ligand of interest for all drug discovery programs. Here, we describe the development and characterization of a cellular assay based on sortilin interaction with the pro domain of proNGF, that can be employed for medium to high throughput screening purposes that can be easily adapted for other ligands and receptors.

## Methods

### Western Blotting

HEK293 cells transfected as described below were plated in 10 cm poly-lysine coated dishes and were 48 h later split to 24 well dishes. 24 h later, cells were lysed in RIPA lysis buffer containing protease inhibitor (50 mM Tris-HCl pH 7.4, 1% NP-40, 0, 25% Na-deoxycholate, 150 mM NaCl, freshly added 1 tablet PhosSTOP™ (Roche) and 1 tablet/50 ml of cOmplete™ Protease Inhibitor Cocktail (Roche)). An assay (Pierce™ BCA Protein Assay Kit, Thermo Fisher Scientific) to determine protein concentration was carried out according to manufacturer’s instructions. 15 µg of total protein were loaded on a 4–12% BisTris gel (NuPAGE™ Novex™ 4–12% Bis–Tris Protein Gels, 1.0 mm, Thermo Fisher Scientific) in MOPS buffer. The proteins were electroblotted to a PVDF membrane. The membrane was incubated in PBS with 5% dry milk powder. Primary antibodies (1:1000 BD Biosciences number 612101, mouse anti-NTR3) were incubated at 4 °C over night, and washed 3 times in PBST. The secondary antibody (1:1000 Peroxidase-Conjugated Rabbit Anti-Mouse P 0260, DAKO) was added in PBS with 5% dry milk powder for 1 h in the dark. The membrane was washed 3 times in PBST and bands were detected using SuperSignal™ West Dura Extended Duration Substrate (Thermo Fisher) according to manufacturer’s instructions and exposed to film.

### Generation of Antibodies Blocking Sortilin Binding to the NGF pro Domain

A synthetic gene coding for the a human sortilin-human IgG1 Fc fusion protein (human sortilin AA 78-756; human lgG1-FC AA104-330) was cloned into pcDNA3.1 and expressed in HEK293 (freestyle system, lnvitrogen). The immunogen, hSortilin-hIgG1 Fc was purified from media by protein-A affinity chromatography and resuspended in PBS, pH 7.2. Anti-human sortilin antibodies were generated through immunization of 5 BALB/c mice using hSortilin-hIgG1 Fc fusion as an immunogen. A single mouse with satisfactory immune response was selected for cell fusion and hybridoma generation. Hybridoma supernatants were screened by ELISA using human sortilin-ECD as coating antigen. A total of eighteen hybridoma cell lines derived from nine parental clones were generated. Hybridomas were initially grown in complete growth medium, DMEM with 10%FBS + antibiotics, and subsequently adapted to CDhybridoma media (lnvitrogen) for expression. Mouse monoclonal antibodies were purified from hybridoma cell culture supernatants by protein-G Sepharose according to standard procedures (GE healthcare).

### Protein Purification

The expression construct for human proNGF was generated by cloning of a synthetic gene (residues 19–241 of P01138 +6 C-terminal His residues, Geneart) in pcDNA 3.1 expression vector. Cultured media (1000 ml) from transient expression in CHO-S cells (ThermoFisher Scientific) was applied to a 5 ml HisTrap column and washed with 20 mM Sodium Phosphate pH 7.4, 1 M NaCl (A buffer). Elution of bound protein was done in a linear gradient to 0.25 M Imidazole in A-buffer over 20 column volumes and a flow of 5 ml/min. Fractions were analysed by SDS-PAGE and pooled based on proNGF content. Finally, the pool was dialysed against 1× PBS (Invitrogen) at 4 °C. Samples are stored at − 20 °C in aliquots. Recombinant human proNGF expressed and purified from *E.coli* was purchased from Alamone labs.

GSTpro was engineered as a fusion of Glutathione S-transferase (GST) merged at the C-terminal of GST to the pro part (19–121) of human proNGF. The construct was cloned into pGEX expression plasmid and used for expression in *E.coli* using the Overnight ExpressT Autoinduction System 1 (Novagen). The cells were harvested, lysed and from the supernatant the GSTpro was purified, using standard Glutathione-Sepharose affinity chromatography. Neurotensin and Neurotensin derived peptides were synthesized by GenScript Biotech.

### Cell Culture for Sortilin Cell-Based Assay

HEK 293 cells were grown in DMEM with 10% fetal bovine serum. They were transfected with plasmids either encoding wild type sortilin, or sortilin with a mutation that renders it endocytosis deficient, or an empty control vector according to manufacturer’s instructions using 20 µg lipofectamine (Thermo Fischer Scientific) with 8 µg DNA on 4.5 × 10^6^ million cells per 6 cm, poly-lysine coated dish. The cells were initially plated into 24-well dishes after transfection. That intermediate step rendered more uniform cell numbers in the 96-well dishes that were used to run the actual assay. 24 h later, cells were split into black opaque-walled, clear-bottom 96 well dishes at 42000 cells in 80 µl medium/well. 23 h after plating into 96 well dishes, cells were treated with 20 or 100 nM humanized anti-sortilin antibodies to be tested for blocking sortilin—NGF pro-domain interaction, or blocking compounds, or control compounds, or neurotensin (positive control), or a scrambled neurotensin peptide (negative control), or a 4mer or 3mer peptide derived from the C-terminal part of neurotensin (positive control), or a reverse 3mer C-terminal peptide of Neurotensin (negative control). 1 h after that treatment, the medium was replaced with 80 µl medium containing the same antibody, compounds or peptides included in the preincubation medium, plus recombinant GSTpro or proNGF (either purified in-house from recombinant HEK cells or derived from an *E.coli* expression system at either 0 nM (negative control), or 50 nM, or, in a few instances at 5 or 10 nM. The respective concentrations are indicated in the figures 45 min after adding GSTpro or proNGF, cells were washed twice with prewarmed PBS and fixed in 4% PFA for 20 min at approximately 20º C.

### Immunocytochemistry

The fixed cells were washed with PBS for 15 min, followed by two 15 min washes with PBS with 0.1% Triton X-100. The cells were then treated with PBS with 10% FBS for 10 min and subsequently incubated with primary antibodies at 4º C overnight as follows: To test expression of sortilin, control wells were stained with an anti-sortilin antibody at a 1:500 concentration in 10% FBS/PBS (Mouse IgG1 Anti sortilin, BD Transduction Laboratories™ number 612101). As some of the sortilin-pro domain blocking antibodies to be tested were mouse-derived, the use of secondary anti-mouse antibodies for immunohistochemical staining needed to be avoided, as further explained in the results section. Thus, in immunohistochemical staining, goat-derived anti-sortilin antibodies (1:800 affinity-purified polyclonal antibody BAF2934; R&D Systems) were used to test the blocking of sortilin-pro interaction by mouse antibodies. Wells to be evaluated for blocking of the sortilin-GSTpro interaction by antibodies were only stained with an antibody against the pro domain of proNGF in 10% FBS at a dilution of 1:1500 (Millipore (N-term) clone EPI318Y, Rabbit Monoclonal Antibody Catalog Number: #04-1142). To stain against GST, a rabbit anti-GST antibody was applied at 1:600 (abcam ab9085).

The following day, wells were washed 3 × 15 min with PBS/0.1% Triton X-100. The secondary antibodies were centrifuged at 13000 g for 2 min before dilution. All antibodies were diluted in PBS/10%FBS with 0.5 µg/ml Hoechst dye and filtered through a Millipore express MC 0, 22 µm syringe-attached Filter Unit. Cells that had been incubated with a mouse-derived anti-sortilin antibody were incubated with an Alexa 594 donkey anti-mouse antibody at a 1:3000 dilution. Wells that had previously been incubated with an anti-proNGF antibody were subsequently incubated with an Alexa 488 donkey anti rabbit (A110034) antibody at a 1:400 dilution. To detect GSTpro, an Alexa488 goat anti-rabbit (ThermoFisher A11034) antibody was applied at a 1:300 dilution. Both secondary antibodies were applied for 1 h in the dark. Cells were then washed 1 × 15 min with PBS + 0.1% Triton X-100, and 2 × 15 min with PBS. Cellular fluorescence was quantified using an array scanner and the “Neuronal Profiler” Bioapplication (Thermo Fisher Scientific) as described below.

Sortilin-GSTpro interaction was also assessed using HEK cell derived cell lines stably expressing sortilin. To that end, normal HEK cells (negative control) or S18 cells were directly plated into 96 well dishes and treated with antibodies or compounds 23 h later as described above.

To further examine whether GSTpro was internalized, rather than only binding to the cell exterior, extracellularly bound ligand was removed by washing cells in PBS acidified to pH 2.0 with HCl supplemented with 0.03 M sucrose and 10% FCS immediately before fixation.

### Evaluation of Sortilin-Mediated GST-pro Uptake Using Automated High Content Screening

Images from 96 well dishes were automatically recorded with a Cellomics ArrayScan VTI HCS Reader (Thermo Scientific) using a 10× microscope objective and the build-in standard autofocus method. Fifteen 1024 × 1024 images in 4 channels (Channel 1 and 2: Filter XF5–Hoechst, to detect nuclei and all cells; Channel 3: XF53-Texas Red to detect, e.g., sortilin-positive cells; Channel 4: Filter XF93 – FITC, to detect cells positive, e.g., for the proNGF pro domain) were recorded per 96 well.

The images were analyzed with the Cellomics assay algorithm NeuronalProfiling.V3.5.

## Results

**Fig. 1:**
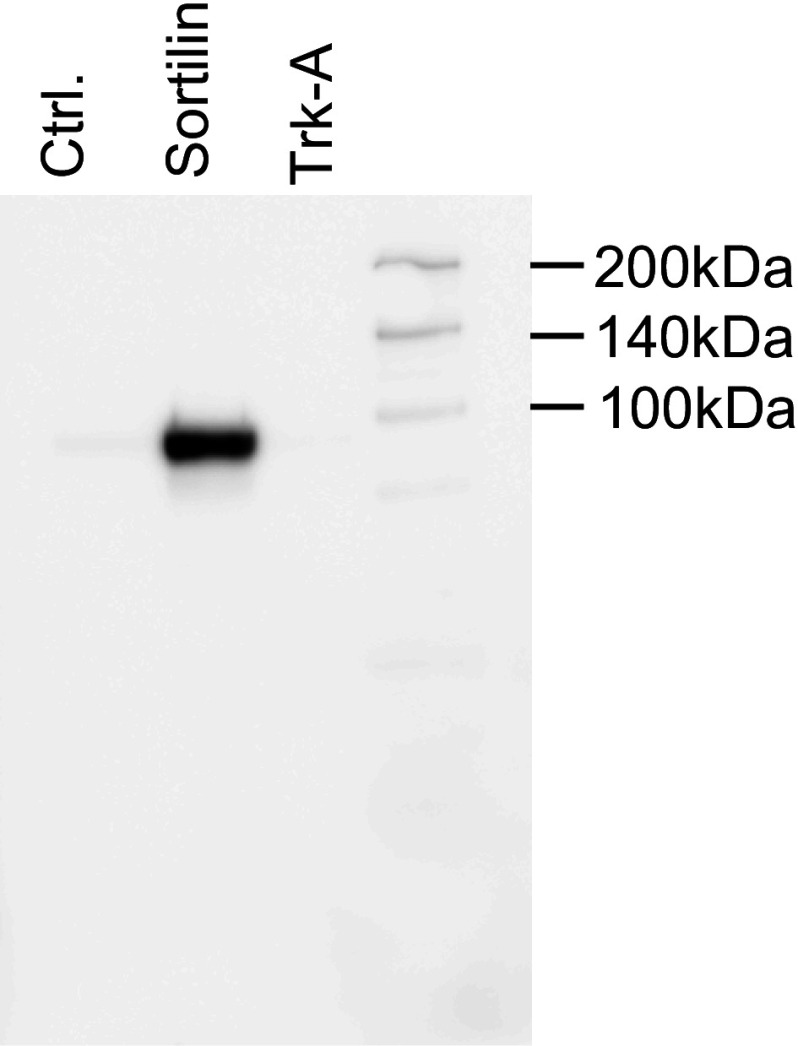
Detection of sortilin in transfected HEK 293 cells by Western blotting. HEK 293 cell lysates from cells transiently transfected with empty vector (Cnt); a Sortilin or a TrkA expression construct were probed on a Western gel using an anti-sortilin antibody

To quantify proNGF uptake mediated by sortilin, we transiently transfected HEK 293 cells with a human *sortilin* expression construct. Sortilin could readily be detected in transfected cells by immunoblotting (Fig. 1, lane 2), while no *sortilin* expression could be detected in cells transfected with empty vector (Fig. 1, lane 1) or in cells transfected with a different receptor (Fig. 1, lane 3). We initially attempted to use commercially available ELISA assays to detect changes in proNGF concentration in the cell culture medium of cells transiently transfected with sortilin. To that end, we tested several commercially available ELISA kits for NGF detection. These assays could readily detect low concentrations of proNGF, but were not sensitive enough to measure the decrease in proNGF concentration due to cellular uptake (data not shown).

**Fig. 2:**
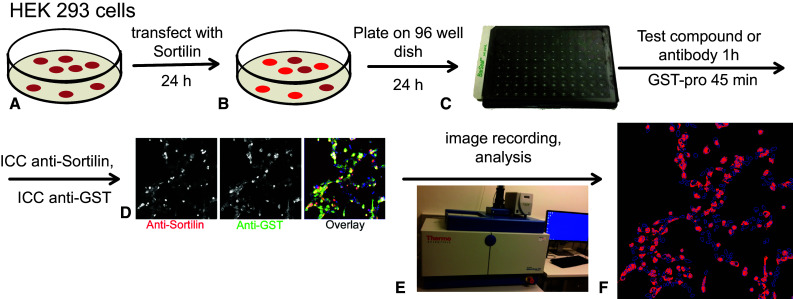
Assessment of pro-NGF pro domain binding to sortilin. **a**. HEK 293 cells were transfected with a sortilin expression construct and incubated for 24 h. **b** Cells were transferred to 96 well dishes and incubated for another 24 h. **c** Cells were treated with test compounds or blocking antibodies for 1 h, followed by treatment with GST-pro for 45 min. **d** Cells were subsequently fixed and stained using anti-sortilin and anti-GST antibodies. **e** Images were automatically recorded using an array scanner. F. Recorded images were analyzed using an automated algorithm. Shown here is sortilin staining gated above a set threshold (red) and nuclei (blue). The “sortilin-negative” cell population with no red staining in their vicinity can be analyzed separately from the “sortilin-positive” cell population. Each result was confirmed by at least 3 independent experiments

The need for a highly sensitive assay to specifically measure proNGF uptake by sortilin on the cell surface lead us to develop a novel cell based assay that can quantify cells that express sortilin and measure binding the pro domain of proNGF (Fig. 2). Quantification of the assay has the benefit of automated image recording (Fig. 2e) and computer-assisted image analysis (Fig. 2f) to increase throughput and reproducibility.

**Fig. 3:**
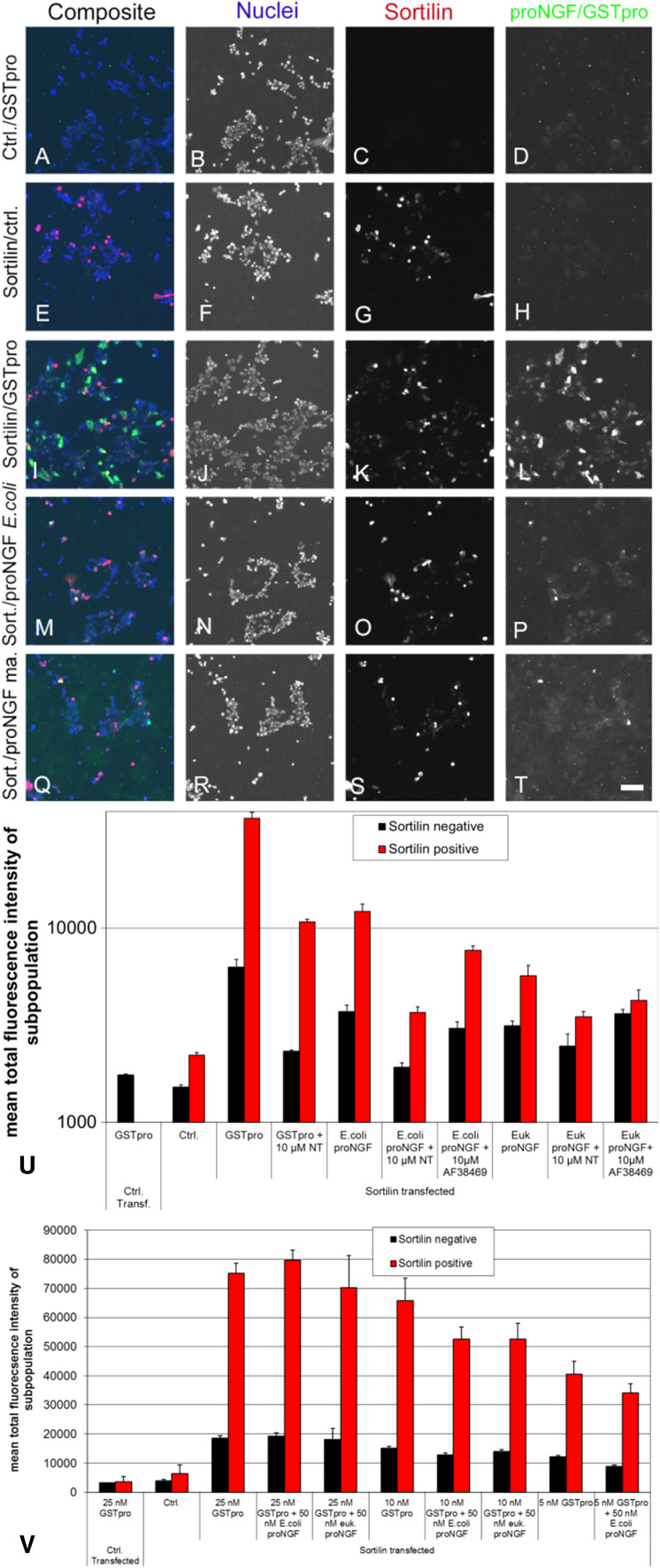
Compared to recombinant pro-NGF, GST-pro renders a larger signal and assay window. A-T Immunohistochemistry on HEK 293 cells using Hoechst staining (nuclei); anti-sortilin staining; or staining directed against the pro domain of proNGF (proNGF/GSTpro); or composite image showing all staining concomitantly as indicated above the panels. **a**–**d** Cells transfected with an empty expression vector and treated with GSTpro. **e**–**h** Cells transfected with a sortilin expression construct, but not treated with GSTpro. **i**–**l** Cells transfected with sortilin and GSTpro treated. **m**–**p**, Cells transfected with sortilin and proNGF purified from recombinant *E.coli*. **q**–**t** Cells transfected with sortilin and proNGF purified from a eukaryotic expression system. Nuclei were visualized by Hoechst staining (B, F, J, N, R). Interaction of the cells with proNGF or with GSTpro was visualized by anti-proNGF, or anti-GST immunofluorescence, respectively. Space bar: 100 µm. **u** Detection of GSTpro and eukaryotically or prokaryotically expressed proNGF binding in sortilin positive and sortilin negative cells by automated analysis. HEK 293 cells were both transfected with empty vector (left-most column pair) or transfected with a sortilin expression construct and treated as indicated. Black columns: Total fluorescence intensity of “sortilin-negative” cells. Red columns: Total fluorescence intensity of sortilin positive cells. Note the logarithmic scale on the vertical axis. **v** Both proNGF isolated from recombinant *E. coli* and proNGF isolated from a mammalian expression system (euk. proNGF) compete against GSTpro binding to wt sortilin. wt sortilin-transfected HEK cells were treated with 5, 10, or 25 nM GSTpro concomitantly with 50 nM proNGF, and subsequently stained for GST

To develop the assay, we initially tested the treatment of sortilin-transfected cells with different recombinant forms of proNGF to evaluate potential impacts of posttranslational modification and sequence context on binding. ProNGF was purified from either *E. coli* expression system (Fig. 3m–p) or form HEK 293 cells (Fig. 3q–t), or as a GSTpro fusion protein with NGF pro domain fused at the GST C-terminus (GSTpro, Fig. 3a–d, i–l). Both proNGF and GSTpro could readily be detected by immunocytochemistry. Sortilin partly colocalized with GSTpro or proNGF (Fig. 3i, m, q). Also, those interactions could be partially blocked by neurotensin or by compound AF38469 (Fig. 3u; also cf. Figure 5l with t and Fig. 5i with q). AF38469 is a compound isolated in a screen to block binding of the NGF pro domain to Sortilin (Schrøder et al. 2014).

The assay was compromised by the tendency of proNGF to form small precipitates outside the cells. This behavior led to more diffuse staining compared to GSTpro. In addition, we showed by ELISA that the proNGF concentration was reduced by merely incubating it in empty cell culture dishes with no cells present confirming the tendency of proNGF to stick to the wells (not shown). Given the complications driven by the biophysical properties of proNGF, we pursued GSTpro as a source of the pro binding domain. The signal and assay window when using GSTpro was substantially larger compared to proNGF (Fig. 3u, note the logarithmic scale). Furthermore, GSTpro is also easier to generate and more stable than proNGF, making GSTpro a potentially cheaper and more reliable reagent.

We evaluated different assay conditions to show that GSTpro was a suitable surrogate for proNGF. First, we showed that proNGF competed with GSTpro for binding to sortilin (Fig. 3v). GSTpro binding to cells was shown to be mediated by sortilin as we detected only very low signal in the empty vector control-transfected cells (Fig. 3a, c, d). To further improve the signal-to-noise ratio, we evaluated the sortilin-GSTpro interaction in both sortilin-positive (red bars, e.g., in Fig. 3u, v) and sortilin-negative cells (black bars, e.g., in Fig. 3u, v). Cells were designated “sortilin negative” (black bars) if the sortilin expression based signal intensity was below a fixed threshold. Those cells may still express sortilin at a low level, which, however, will not influence the evaluation of the cells defined to be “sortilin-positive” (red bars). In cells transfected with an empty vector (for instance, Fig. 3a, c; Fig. 3u, v left-most column pair) or cells not treated with either proNGF or GSTpro (for instance, Fig. 3e, h, u, v), very low fluorescence background signal was detected.

**Fig. 4:**
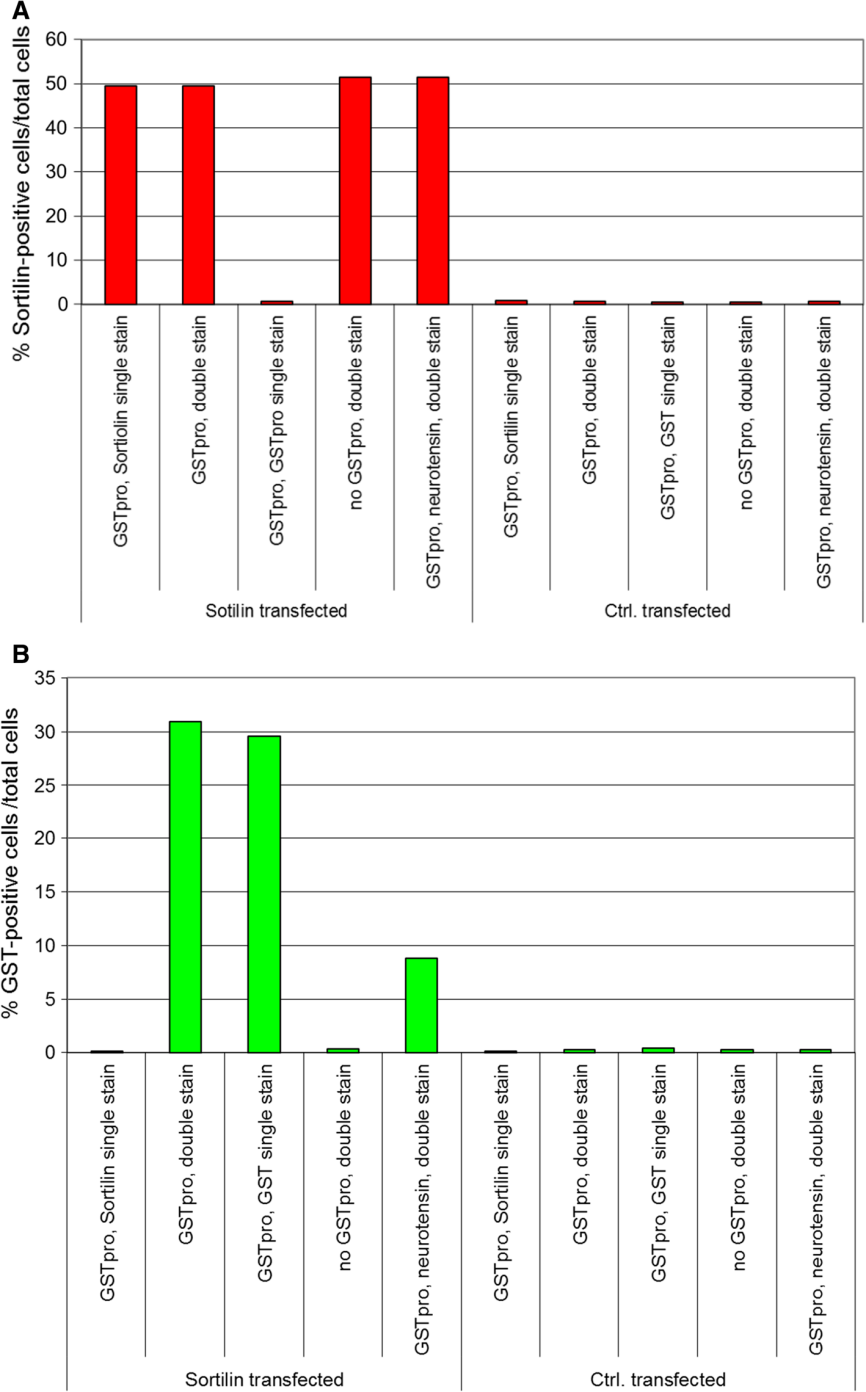
Control values determined in each experiment to ensure specificity of the detection procedure. **a** Demonstration of high specificity of automated detection of sortilin positive cells. Cells were either sortilin transfected (left 5 columns) or transfected with empty vector (5 right-most columns). 48 h later, cells were either incubated with GSTpro or with solvent (no GSTpro) for 45 min as indicated. One well each was also incubated with the peptide neurotensin, blocking sortilin-pro-domain interaction (column 5, 10). Subsequently, cells were either immunostained for sortilin, or GST, or for both (double stain). All immunocytochemical reactions were incubated with both secondary antibodies, further suggesting specificity of staining.** b** Control values demonstrating highly specific automated detection of pro-GST positive cells. Cells were treated as indicated in figure legend a

We evaluated the specificity of the staining and the level of fluorescent signal leakage between different filter sets. We performed single staining against either sortilin or GST, to evaluate leakage of fluorescent signal from one microscopic channel to the other channel (Fig. 4). These controls were conducted in all experiments to confirm the robustness of the data. Sortilin-expressing cells were specifically detected (Fig. 4a, cf. column 1, 2, 4, 5 with column 3 [single staining against GST-pro, no anti-sortilin staining] and with columns 6–10 [cells not sortilin transfected]). In not transfected HEK cells, consistently less than 1% of cells were detected as sortilin positive. Likewise, staining against the ligand was also specific (Fig. 4b, cf. columns 1 [only staining for sortilin] and 4 [Sortilin-GSTpro double staining, but no GSTpro treatment] with columns 2, 3). In cells not stained against GSTpro or not GSTpro treated, less than 0.5% of the cells were detected as false ligand positive. Even when not separately evaluating the sortilin-positive and sortilin-negative subpopulations of cells, it was obvious that transfection with sortilin highly enhanced GSTpro uptake or binding (Fig. 4b, cf. column 2 and 3 [sortilin transfected cells] with columns 7 and 8 [cells transfected with control vector]).

**Fig. 5:**
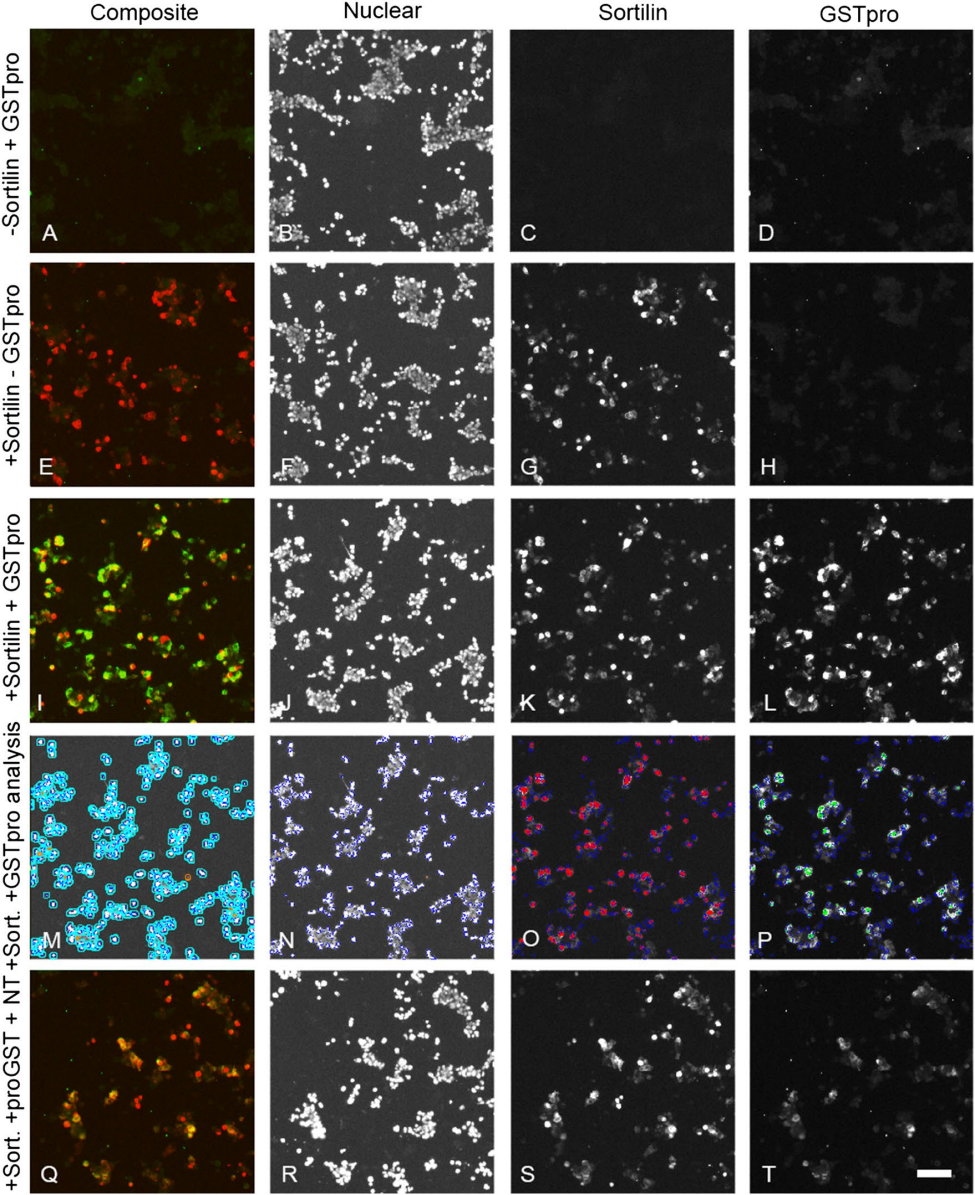
Sortilin-expressing HEK293 cells display enhanced GSTpro binding, which can be partly inhibited by Neurotensin. All cultures shown were fluorescently co-stained using antibodies against GST and sortilin.** a**,** e**,** i**,** q**: Figure composed of combined anti-sortilin (red) and anti-GST (green) staining.** b**,** f**,** j**,** n**,** r**: Hoechst staining.** c**,** g**,** k**,** s**: Anti-sortilin staining.** d**,** h**,** l**,** t**: Anti-GST staining.** a**,** b**,** c**,** d**: HEK cells not transfected with sortilin.** e**,** f**,** g**,** h**: Cells transfected with sortilin, but not treated with GSTpro.** i**,** j**,** k**,** l**, cells transfected with sortilin and treated with GSTpro.** m**, Automated image analysis of the image in panel** j**., defining an “area of interest” around each cellular nucleus.** n**, Automated Identification of nuclei of the image shown in** j** by the image analysis algorithm.** o** Automated image analysis of the image shown in** k**, identifying anti-sortilin staining above threshold (red).** p** Automated image analysis of the image shown in** l**, identifying anti-GST staining above threshold (green).** q**,** r**,** s**,** t**: Cells transfected with sortilin and treated with neurotensin and GSTpro. Space bar in panel T: 100 µm

The control experiments suggest that automatic GSTpro detection on sortilin-transfected HEK cells could be used to evaluate interference of novel compounds with sortilin—NGF pro domain interaction. We tested neurotensin, a known sortilin ligand. We showed it substantially reduced sortilin-GSTpro binding (Fig. 5b, cf. panels i, l with panels q, t, respectively).

**Fig. 6:**
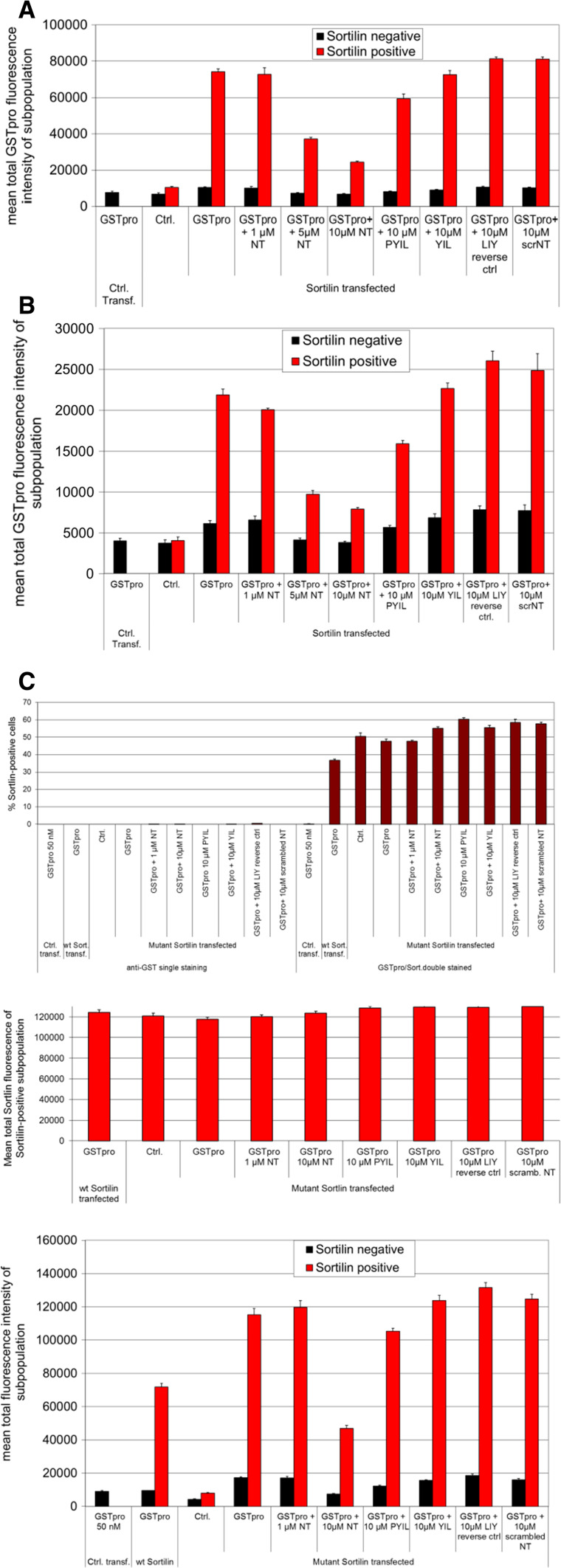
Assessment of binding of the NGFpro domain to sortilin-negative and sortilin-positive cells. **a** Sortilin-positive cells (red bars), compared to sortilin-negative cells (black bars), display a stronger GSTpro fluorescence signal, which can be blocked by neurotensin and neurotensin-derived peptides, but not by control peptides. Cells were both transfected with vector without insert and GSTpro-treated (left-most column); sortilin-transfected, but not GST-pro treated (second column pair); or sortilin transfected and treated as indicated below each column pair. (NT: neurotensin; PYIL: neurotensin-derived C-terminal 4mer peptide; YIL: neurotensin-derived C-terminal 3mer peptide; LIY: reverse neurotensin-derived 3mer peptide; scrNT: peptide with a scrambled neurotensin peptide. **b** In a similar experiment as shown in a, the GSTpro fluorescent signal almost fourfold reduced, when immunocytochemical staining is performed without permeabilization of cells with detergent. **c** Compared to wt sortilin expressing cells, internalization-mutant sortilin expressing cells show over 60% more GSTpro signal. Top graph: Data demonstrating specificity of sortilin-positive cell detection and slightly fewer cells expressing wt compared to internalization-mutant sortilin. Under no condition were substantial numbers of false sortilin-positive cells detected when cells were stained for anti-GSTpro only (10 left-most columns); or when cells were transfected with empty vector (Ctrl. transf., 11th column). The total fluorescence intensity from wt sortilin-transfected cells (12th column) was similar to the signal from internalization-mutant sortilin-transfected cells (8 right-most columns). Middle graph: In those cells that are sortilin-positive, total average sortilin fluorescence per cell is similar under all conditions, suggesting equal expression levels, but somewhat lower transfection efficiency of the wt construct compared to the mutant plasmid. Lower graph: Cells were both transfected with a control vector with no insert (left column pair); wt sortilin (second from left column pair); or transfected with an internalization-mutant sortilin and treated with other agents as indicated and explained in **a** and **b**. Abbreviations: NT: Neurotensin; PYIL: 4mer peptide derived from the C terminal of NT; YIL: 3mer peptide derived from the NT C-terminal; LIY: 3mer NT-derived peptide with amino acids in reverse order; scrNT: NT peptide with amino acids in a scrambled order

The ligand signal was to a lesser extent also reduced by Neurotensin C-terminally derived 4mer (PYIL) peptide, but not with a 3mer (YIL) peptide, or such a 3mer peptide with amino acids in a reversed order (LIY) or with a peptide that contained Neurotensin amino acids in a scrambled order (Fig. 6a), suggesting that the competition with sortilin-GSTpro binding was specific. The calculation for mean fluorescence was performed using an algorithm that measures pixel intensity of all pixels within the area of interest (regions the program identified as cells), integrates the values for all pixels and calculates the mean for all cells of the subpopulation. Thus, the assay generates a measurement of inhibition based on a subpopulation of cells that express defined levels of sortilin and quantifiable levels of ligand bound on the cell surface.

It was not clear whether the detected ligand signal was only due to GSTpro binding to sortilin, or whether sortilin would be subsequently internalized. To determine if GSTpro was endocytosed after sortilin binding, we performed immunocytochemical staining with and without detergent (Fig. 6a, b, respectively). Omitting a permeabilization step in the staining procedure reduced the GSTpro signal almost fourfold in sortilin positive cells (red bars), while it was less than half reduced in cells expressing sortilin below the set threshold. These data are consistent with a large fraction of the GSTpro signal being derived from internalized ligand (cf. absolute values in Fig. 6a with b). However, alternative interpretations are also possible (see discussion). In the absence of permeabilization (w/o detergent), neurotensin and neurotensin-derived peptides still competed with the GSTpro-sortilin interaction. We investigated if GSTpro was bound to the cell surface by using an acid wash to remove surface bound GSTpro. Cells were washed in PBS, pH 2.0 immediately before fixation, to remove extracellular ligand bound to receptors. This reduced the GST-pro signal by 20% (data not shown), further suggesting that most of the ligand was internalized upon binding.

To examine whether internalization of the protein was a prerequisite of sortilin- GSTpro interaction, we performed the assay using an internalization-mutant sortilin (Fig. 6c). The fraction of cells expressing mutant sortilin above the arbitrary threshold set for sortilin detection was higher compared to those cells detected after wt sortilin transfection (Fig. 6c, upper graph, column 13 corresponds to a 38% higher value compared to column 12). However, in transfected cells, expression levels between wt and mutant sortilin expressing cells did not differ (Fig. 6c, middle graph, cf. left-most column with the other columns). These results suggest that transfection efficiency was higher with the mutant construct and the expression levels of sortilin in cells that were successfully transfected with either mutant or wt was similar. Compared to wt sortilin transfected cells, mutant sortilin transfected cells showed an approximately 60% higher GSTpro signal (Fig. 6c bottom graph, compare column pair 2 with column pair 4). Interestingly, cells expressing the internalization mutant were less sensitive to neurotensin blocking of the sortilin-GSTpro interaction. In contrast to wt sortilin transfected cells, a concentration of 1 µM neurotensin was not sufficient to reduce the GSTpro signal (Fig. 6c. bottom graph, column pair 5).

**Fig. 7:**
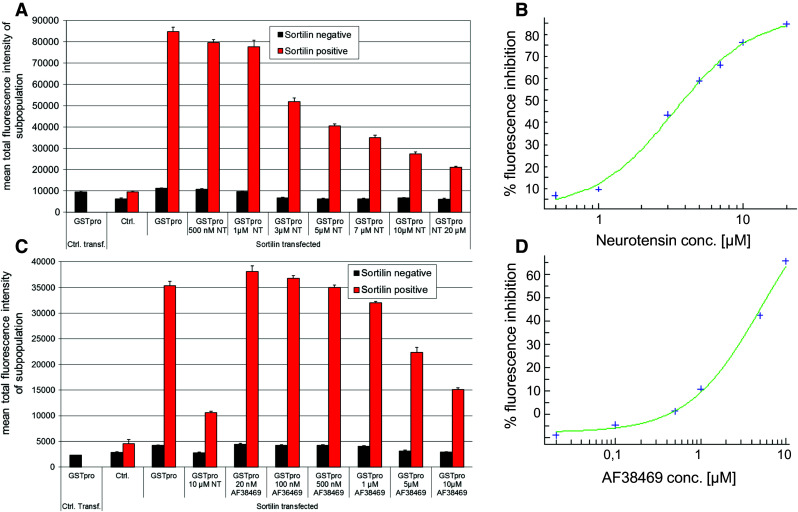
Blocking of sortilin-pro-domain interaction by neurotensin or compound AF38469 is dose-dependent. **a** Cells were both transfected with vector without insert and GSTpro-treated (left-most column); sortilin-transfected, but not GST-pro treated (second column pair); or sortilin transfected and treated as indicated below each column pair (NT: neurotensin). **b** Inhibition of GSTpro fluorescence in sortilin-positive cells (red bar in a) by neurotensin. **c** Cells were treated similarly as described in Figure a, but compound AF38469 was used instead of Neurotensin at the indicated concentrations. **d** Inhibition of GSTpro-sortilin interaction in sortilin-positive cells by compound AF38469

To further characterize the dose-dependency of inhibition of sortilin-GSTpro interaction by neurotensin, we treated cultures with neurotensin concentrations between 0.5 to 20 µM 1 h before applying GSTpro (Fig. 7a, b). Neurotensin dose-dependently inhibited GST-pro binding to sortilin, with an IC_50_ for sortilin-positive cells of over 3 µM. We performed a similar experiment with compound AF38469, which dose-dependently blocked sortilin-mediated GST-pro binding with an IC_50_ of over 5 µM (Fig. 7c, d).

**Fig. 8:**
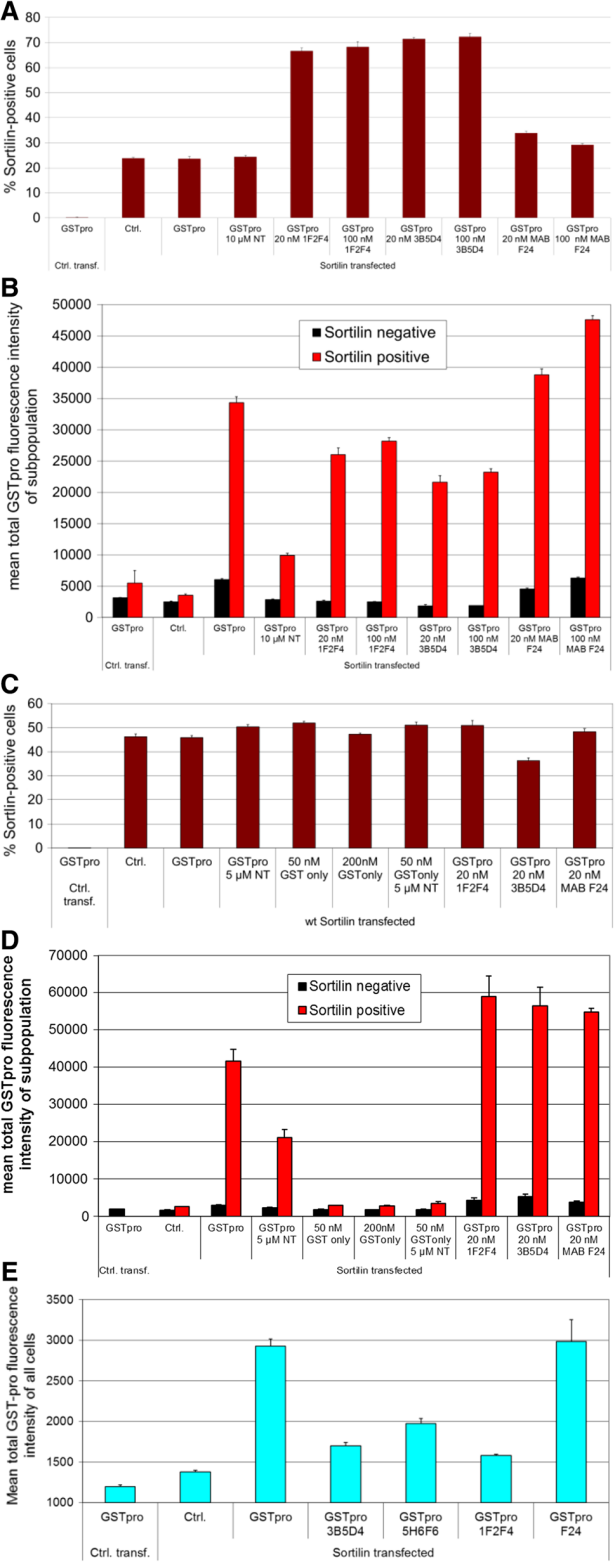
Detection of antibody interference with sortilin-GSTpro interaction. HEK293 cells were both transfected with empty vector (Ctrl. transf.) or with a sortilin expression construct, and either not treated (Ctrl.), or treated with GSTpro, with or without neurotensin (NT) as a positive control, or the indicated antibodies. **a** Treatment of the cultures with mouse-derived monoclonal anti-sortilin antibodies increases the apparent fraction of sortilin-positive cells, as for detection of sortilin-positive cells mouse IgG1 antibodies were used. **b** Antibodies 1F2F4 and 3B5D4, but not antibody F24 decrease the GSTpro signal in sortilin-positive cells even when mouse-IgG1 anti-sortilin antibodies are used for detection. **c** Using a goat anti-mouse-sortilin antibody in the immunocytochemical staining procedure, the undesirable increased sensitivity of sortilin detection after treatment of the cultures with anti-sortilin antibodies is ameliorated **d** When a goat-derived anti-sortilin antibody is used for immunofluorescent sortilin detection, blocking of sortilin-GSTpro interaction with anti-sortilin antibodies cannot be detected. GST only: GST without the pro domain as a negative control in fig **c** and **d**. **e** Without concomitant immunofluorescent anti-sortilin staining, blocking of sortilin-GSTpro interaction can readily be detected in the whole cell population, of course precluding separate detection in the sortilin-positive and sortilin-negative cell population

We also tested the effect of antibodies generated to block sortilin-GSTpro binding. The antibodies used were derived from immunization of mice with human sortilin (materials and methods). Cultures treated with the mouse anti-sortilin antibodies showed an almost threefold increase in the apparent fraction of sortilin-positive cells (Fig. 8a, cf. column 2, 3, 4 with columns 5–10). This was likely due to binding cells with a low expression of sortilin, which were previously not detected as sortilin-positive in the control conditions. The cross reactivity with the anti-sortilin blocking antibody was high affinity and, therefore, not removed by the subsequent washing steps applied by the immunocytochemical procedure. Although partial blocking of the sortilin-GSTpro interaction by some of the testing antibodies could be detected (Fig. 8b, column pairs 5–8), certainly the altered apparent fraction of sortilin-positive cells may skew the readout and prevent an accurate quantification of the blocking efficiency. Moreover, the assay window was small, and the blocking was not dose dependent between 20 or 100 nM of anti-sortilin antibody. We therefore established the assay using a goat-derived anti-sortilin antibody in the immunocytochemical procedure. As expected, the use of different secondary antibodies for sortilin detection ameliorated the apparent increase in sortilin positive cells (Fig. 8c). However, unexpectedly, treatment of the cultures with those antibodies leads to an apparent increase in the GSTpro signal (Fig. 8d). We suspected that somehow the double staining procedure interfered with the GSTpro signal readout. We, therefore, omitted the staining against sortilin when antibodies were tested in the assay, which of course precluded the separate evaluation of sortilin-positive and sortilin-negative cells. Using that procedure, blocking of the sortilin-GSTpro interaction could readily be detected (Fig. 8e). Many of the results presented here were also confirmed using a cell line stably expressing sortilin (data not shown).

## Discussion

The goal of this study was to develop a cell-based assay to screen compounds for their ability to block sortilin binding to the NGF pro domain. The current assay allows specific detection of binding competition for NGF pro domain binding to cell surface expressed sortilin. This assay format provides data that are biologically relevant compared to just assessing in vitro binding of a compound to sortilin, e.g., bound to a matrix.

The assay format confirmed that GSTpro is endocytosed upon receptor-ligand binding, as staining without permeabilizing the cells renders a much lower GSTpro signal (Fig. 6a, b). Another interpretation of that result is that staining of the cells is overall more efficient when detergent is included in the staining procedure. Consistent with sortilin-mediated endocytosis of GSTpro upon binding, acid washing of the cells only removes a small fraction of the GSTpro signal. This is consistent with earlier work on endocytosis of ligand bound sortilin that has been reported for neurotensin (Mazella et al. 1998; Navarro et al. 2001). However, it should be pointed out that internalization is not a prerequisite for this assay as internalization deficient mutant of sortilin results in a stronger GSTpro signal than wild type sortilin (Fig. 6a, c).

Due to reasons of stability of the proNGF protein and the specificity of binding, we recommend use of GSTpro in the assay, rather than recombinant proNGF. We detected unexpected binding of proNGF (pI = 10) to polylysine coated culture plates possibly due to the unstable nature of the protein. Although not formally tested by us, there may have been inconsistencies in the batches of proNGF protein used in the studies. As extracellular fluorescence could increase false positive cells by automated detection, proNGF was less reliable as a reagent in our assay (not shown). Both GSTpro and proNGF compete for binding to the sortilin receptor, it is likely that their binding sites are similar or at least overlap. Detection with GSTpro was more specific than with proNGF. Furthermore, it is more stable and has an overall lower production cost. The assay has the potential to use a tenfold lower concentration of GSTpro (5 nM, cf. Fig. 6v).

Consistently, there was a several fold higher GSTpro signal in sortilin-positive cells compared to sortilin-negative cells (cf. red bars to black bars in Figs. 3, 6, 7, 8). This method analyzes subpopulations of cells based on sortilin expression levels for GSTpro binding by use of an array scanner combined with high-content analysis. This enables evaluation of GSTpro binding to sortilin on cells expressing sortilin at a high level separately from cells expressing sortilin below a fixed threshold. In our studies, the threshold was selected in a way that the negative control for sortilin expression, vector control-transfected cells, would display less than 0.5% sortilin positive cells. That procedure increased the assay window and allowed to specifically examine those cells relevant for the scientific question examined.

We observed that the sortilin staining reagents interfered with the detection of GSTpro binding in the presence of sortilin-GSTpro blocking antibodies. This interference was detected independent of whether the blocking of the sortilin-GSTpro binding influenced the detection of sortilin-positive cells. The interference could be due to the presence of both the sortilin staining and blocking antibodies bound to sortilin on the cell surface simultaneously. The presence of both antibodies could impact the binding of GSTpro to sortilin indirectly or trap GSTpro on the surface in a nonspecific fashion. We assessed antibody-mediated blocking of sortilin-GSTpro binding by single staining against GSTpro only, evaluating GSTpro binding in the whole cell population rather than in the sortilin-positive cell population. In the absence of sortilin staining, the assay has a reduced sensitivity and a narrower window of resolution compared to of the use of dual staining of sortilin expressing cells and GSTpro used for small molecules and peptides. Regardless of that fact, it detected blocking of sortilin—GSTpro binding.

Previously, neurotensin has been reported to reversibly bind to lysates of sortilin transfected cells with an affinity of 10–15 nM (Mazella et al. 1998). For immobilized sortilin, affinities of 5 nM and 8 nM have been reported for proNGF and GSTpro (Nykjaer et al. 2004). Consistent with those results, we report that comparatively high concentrations of neurotensin are required to compete with GSTpro. Neurotensin dose-dependently blocked sortilin GSTpro binding with an IC_50_ over 3 µM. The assay was also used to screen for blocking of sortilin-GSTpro interaction with compound AF38469, which could readily be detected. Thus, the assay is useful to quantify inhibition of sortilin binding to the NGF pro domain by peptides and small molecules. However, to assess blocking of the interaction by antibodies, staining of the cultures for sortilin and subsequent separate evaluation of GSTpro binding to sortilin positive and sortilin negative cells is not recommended. Rather, as explained above, for that application, staining against sortilin should be omitted; the interaction should be measured using the entire cell population.

A reduction in the GSTpro signal by test agents can be interpreted in different ways. One explanation is interference with sortilin-GSTpro binding detection. A potential mechanistic explanation is that receptor engagement by peptides, antibodies or chemical entities leads to receptor internalization. This would lead to a reduction of receptor present on the cellular surface and fewer sortilin—GSTpro complexes would be observed. Surface expression of an internalization-deficient mutant form of sortilin showed results similar to wt sortilin, suggesting that it is indeed blocking of the sortilin-GSTpro interaction on the cell surface that we detect in our assay. The slightly higher signal for GSTpro in the mutant compared to wt sortilin transfected cells, in spite of same expression levels in those cells that are sortilin-positive, may be due to the mutant receptor being more accessible on the cellular surface, thus being able to bind more ligand per cell compared to wt sortilin.

Very similar results were obtained when HEK293 cells were used that stably expressed sortilin (data not shown). However, we routinely used transiently sortilin-transfected HEK cells, as those allowed us to include a control of HEK cells transfected with vector without insert.

In our assay, we have routinely used a concentration of 50 nM GSTpro, as such concentration was used in many published in vitro experiments involving proNGF. These concentrations are not pharmacologically relevant, as these high levels of neurotrophins are not normally reached in the brain or in the periphery. Thus, likely much lower concentrations of sortilin blocking compounds would likely be required in vivo to achieve a receptor occupancy that would cause biologic effects. Whether the effects observed in vitro can be related to a pharmacodynamic effect in vivo remains to be determined. The assay described here provides a valuable means to identify compounds or peptides as tools to further examine sortilin function. Further preclinical evaluation using primary cell systems and animal models will need to be employed to determine if these compounds have any potential therapeutic application.

Other sortilin ligands could also be used for binding studies to identify inhibitors. One such ligand is the secreted growth factor, Progranulin (PGRN), which endocytosis is also mediated by sortilin (Hu et al. 2010; Zheng et al. 2011). Thus, an assay could also be based on PGRN binding and functional readouts. However, as we were interested in blocking the binding of proNGF to sortilin, such an assay would not have been suitable: ProNGF is reported to bind to the p75NTR/Sortilin complex with high affinity, whereas PGRN shows no affinity for p75^NTR^ alone or in complex with sortilin (Hu et al. 2010). However, depending on the application, a modification of the assay in which both sortilin and p75^NTR^ are overexpressed may be considered. Depending on the goal of the drug discovery campaign, a functional readout is desirable, such as apoptosis induced in cells overexpressing both sortilin and p75^NTR^. An assay that used FRET-based methodology to assess sortilin—p75^NTR^ interaction has recently been published, which provides another very useful approach to be used in screening efforts (Skeldal et al. 2015).

The substitution of proNGF with GSTpro provided us with a more reliable reagent to enable throughput and robustness of the assay. While the assay maintains important parameters of the receptor-ligand interaction, the context of the interaction does not fully mimic the endogenous biological setting. The requirement for retaining the natural context of the ligand may be important to recapitulate the endogenous activity of the ligand-receptor interaction; in these circumstances, a surrogate may not be sufficient. In some cases, the use of cell lines other than HEK cells may be necessary. For instance, to support specific applications in neurodegenerative diseases, the use of neuronal cells coexpressing p75^NTR^ and a functional readout may be required. The value of the assay system described is that it serves as a primary screening tool to allow selection of a limited number of candidates which could then be further characterized using primary cell culture systems and ultimately analyzed in animal models.

An important advantage is the assay specifically evaluates those cells that express the target receptor above a set threshold, with an internal control in each cell culture well provided by the cells that do not express the receptor, or express it at a low level. It will facilitate the optimization of entities that inhibit sortilin interaction with its ligands. It is suitable for medium-throughput screening applications. With few modifications, it can be applied to screen for interference with additional sortilin ligands and even to accommodate other receptors that are targets for pharmaceutical intervention.

## Abbreviations

BDNF: Brain-derived neurotrophic factor
CHO: Chinese hamster ovary
FCS: Fetal calf serum
GST: Glutathione-S-transferase
GSTpro: GST C-terminally linked to the pro domain from NGF
HEK: Human embryonic kidney
NGF: Nerve growth factor
NTR: Neurotrophin receptor
proNGF: NGF precursor protein
